# Reducing arthritis fatigue impact: two-year randomised controlled trial of cognitive behavioural approaches by rheumatology teams (RAFT)

**DOI:** 10.1136/annrheumdis-2018-214469

**Published:** 2019-02-06

**Authors:** Sarah Hewlett, Celia Almeida, Nicholas Ambler, Peter S Blair, Ernest H Choy, Emma Dures, Alison Hammond, William Hollingworth, Bryar Kadir, John Richard Kirwan, Zoe Plummer, Clive Rooke, Joanna Thorn, Nicholas Turner, Jon Pollock

**Affiliations:** 1 Department of Nursing and Midwifery, University of the West of England Bristol, Bristol, UK; 2 Pain Management Centre, Southmead Hospital, Bristol, UK; 3 Department of Population Health Sciences, University of Bristol, Bristol, UK; 4 Section of Rheumatology, Division of Infection and Immunity, Cardiff University, Cardiff, UK; 5 Centre for Health Sciences Research, School of Health Sciences, University of Salford, Salford, UK; 6 Department of Translational Health Sciences, Academic Rheumatology, University of Bristol, Bristol, UK; 7 Patient Research Partner, Academic Rheumatology, Bristol Royal Infirmary, Bristol, UK; 8 Bristol Randomised Trials Collaboration, Bristol Trials Centre, University of Bristol, Bristol, UK; 9 Department of Health and Social Sciences, University of the West of England Bristol, Bristol, UK

**Keywords:** rheumatoid arthritis, fatigue, cognitive behavioural therapy, randomised controlled trial

## Abstract

**Objectives:**

To see if a group course delivered by rheumatology teams using cognitive-behavioural approaches, plus usual care, reduced RA fatigue impact more than usual care alone.

**Methods:**

Multicentre, 2-year randomised controlled trial in RA adults (fatigue severity>6/10, no recent major medication changes). RAFT (Reducing Arthritis Fatigue: clinical Teams using CB approaches) comprises seven sessions, codelivered by pairs of trained rheumatology occupational therapists/nurses. Usual care was Arthritis Research UK fatigue booklet. Primary 26-week outcome fatigue impact (Bristol RA Fatigue Effect Numerical Rating Scale, BRAF-NRS 0–10). Intention-to-treat regression analysis adjusted for baseline scores and centre.

**Results:**

308/333 randomised patients completed 26 week data (156/175 RAFT, 152/158 Control). Mean baseline variables were similar. At 26 weeks, the adjusted difference between arms for fatigue impact change favoured RAFT (BRAF-NRS Effect −0.59, 95% CI –1.11 to -0.06), BRAF Multidimensional Questionnaire (MDQ) Total −3.42 (95% CI –6.44 to -0.39), Living with Fatigue −1.19 (95% CI –2.17 to -0.21), Emotional Fatigue −0.91 (95% CI –1.58 to -0.23); RA Self-Efficacy (RASE, +3.05, 95% CI 0.43 to 5.66) (14 secondary outcomes unchanged). Effects persisted at 2 years: BRAF-NRS Effect −0.49 (95% CI −0.83 to -0.14), BRAF MDQ Total −2.98 (95% CI −5.39 to -0.57), Living with Fatigue −0.93 (95% CI −1.75 to -0.10), Emotional Fatigue −0.90 (95% CI −1.44, to -0.37); BRAF-NRS Coping +0.42 (95% CI 0.08 to 0.77) (relevance of fatigue impact improvement uncertain). RAFT satisfaction: 89% scored > 8/10 vs 54% controls rating usual care booklet (p<0.0001).

**Conclusion:**

Multiple RA fatigue impacts can be improved for 2 years by rheumatology teams delivering a group programme using cognitive behavioural approaches.

**Trial registration number:**

ISRCTN52709998.

**Key messages**

**What is already known about this subject?**

- Fatigue in rheumatoid arthritis is common and cognitive behavioural therapy can help, but few rheumatology units have clinical psychologists to deliver it.

**What does this study add?**

- This study demonstrates that rheumatology nurses and occupational therapists using cognitive behavioural approaches can reduce fatigue impact with both short-term and long-term effects.

**How might this impact on clinical practice or future developments?**

- All seven clinical teams were able to deliver this intervention, suggesting future clinical implementation is feasible.

## Background

Fatigue is a significant problem for people with rheumatoid arthritis,^1^ incorporating physical exhaustion and cognitive impairment, with impacts on lifestyle, roles, relationships and emotions.^2 3^ RA fatigue is persistent,^4^ patients feel unsupported by rheumatology teams and rheumatology nurses want help regarding fatigue management.^2 5^ The RA fatigue causal pathway remains unclear.^6^ Multiple factors may form different combinations and weightings for each patient at each episode. A conceptual framework proposes three main components: inflammation (directly or through pain, sleep disruption, disability), personal factors (eg, work, comorbidities) and cognitive behavioural elements (under/over activity, driven by thoughts/feelings).^7^ Meta-analysis of randomised controlled trials (RCTs) reporting RA fatigue shows small-moderate effects from biological disease modifying anti-rheumatic drugs and physical exercise, although fatigue was rarely the primary outcome.^8 9^

Meta-analysis of psycho-educational interventions reporting RA fatigue also shows small-moderate benefit.^10^ However, only two specifically addressed RA fatigue.^10 11^ One intervention targeted distress, comprising 1–1 cognitive behavioural therapy (CBT), with an optional fatigue module.^10^ The second intervention aimed to reduced fatigue impact, using group CBT.^11^ Both improved fatigue/fatigue impact but were delivered by clinical psychologists, meaning they are hard to implement in the current NHS as few rheumatology teams include clinical psychologists.^12^ The aim of this trial was to see if usual care plus a group course delivered by rheumatology teams using cognitive-behavioural approaches (CBA) reduced RA fatigue impact more than usual care alone.

## Methods

The detailed methods have been published^13^ and a summary is provided here.

### Trial design

UK, 7-centre randomised controlled trial of group CBA for fatigue self-management plus usual care (1–1 fatigue information) versus usual care alone.

### Participants

Eligible patients were aged >18 years with confirmed RA^14^ and fatigue severity >6/10 on a Numerical Rating Scale (NRS),^15^ which they considered recurrent or persistent. Exclusion criteria were recent changes to glucocorticoids (6 weeks) or major RA medication (16 weeks) or insufficient English to participate in group discussions. Patients were approached in clinic or by mailshots to departmental databases. Ethics approval was obtained (East of England Norfolk 13/EE/0310), the trial registered (ISRCTN52709998) and patients gave written informed consent.

### Interventions

RAFT is the group course for RA fatigue using CBT approaches previously facilitated by a clinical psychologist.^11^ RAFT comprises seven sessions (weeks 1–6 for 2 hours, consolidation week 14 for 1 hour), addressing behaviours likely to be related to fatigue and their underpinning thoughts and feelings (see online supplementary data), as previously published.^13^ Tutors use exploratory questioning, goal-setting and peer-support to enhance self-efficacy (belief that you can succeed with an activity), prompting changes in self-management.^16 17^ RAFT was manualised for codelivery by pairs of rheumatology nurses/occupational therapists, who trained together over 4 days and delivered an observed course locally before the RCT.^13^

10.1136/annrheumdis-2018-214469.supp1 Supplementary data

Usual care was the Arthritis Research UK fatigue self-management booklet, based on the original RAFT intervention,^18^ provided to and discussed with each patient for approximately 5 min by the research nurse at the baseline visit (ie, pre-randomisation).

### Outcomes

The primary outcome was fatigue impact at 26 weeks, collected by the central trial team by telephone using the Bristol RA Fatigue Numerical Rating Scale (BRAF-NRS Effect).^15^ Other fatigue elements were severity, coping (BRAF-NRSs) and overall impact (BRAF Multidimensional Questionnaire, BRAF-MDQ).^15^ Clinical status comprised pain (NRS), disability (Modified Health Assessment Questionnaire, MHAQ),^19^ sleep (item from Pittsburgh Sleep Quality Index),^20^ disease activity (DAS28^21^ at weeks 0 and 26, replaced at other time-points by self-report (sPDAS2),^22 23^ mood (Hospital Anxiety and Depression scale, HADS),^24^ quality of life (global question, Arthritis Impact Measurement Scale, AIMS)^25^ and leisure activities (discretionary activity subscale, Valued Life Activities, VLA).^26^ Self-efficacy and helplessness underpinning processes were assessed (RA Self-Efficacy scale (RASE), Arthritis Helplessness Index (AHI)).^27 28^ Outcomes were measured at weeks 0, 6, 26, 52, 78 and 104. At weeks 10 and 18, fatigue was measured for exploratory analysis of the week 14 consolidation session. Satisfaction with RAFT/booklet (26 weeks) and social contact (weeks 52, 104) comprised unvalidated questions. Economic and qualitative evaluations will be reported elsewhere.

### Sample size

With 90% power and a two-sided significance of 0.05, 73 patients/arm would detect 1.46 units difference in fatigue impact (effect size 0.54), equal to 75% of the 1.95 units difference achieved by a CBT therapist (SD 2.7, effect size 0.77).^11^ Potential for clustering effects from groups/tutors increased this to 75/arm and allowing for 2 year attrition (50%) we aimed to recruit 150/arm.^29^

### Randomisation

Seven centres each recruited four trial cohorts over 2 years. Once a centre recruited 10–16 participants (cohort), informed consent and baseline assessments were conducted. Computer-generated randomisation for that cohort was performed by the Bristol Randomised Trials Collaboration; thus, randomisation was stratified by centre, and within centres by cohort (1-4). Allocation was 1:1 with the RAFT arm receiving the additional patient if numbers were uneven. Patients randomised to RAFT but unable to attend their course were offered subsequent courses. All patients eligible at screening were accepted, even if they had become ineligible by baseline (medication/fatigue changes) while awaiting full cohort recruitment, mirroring the pragmatic nature of chronic illness interventions.

### Blinding

RAFT group courses meant blinding of patients and clinicians was impossible, but data were analysed blind to arm allocation.

### Statistical methods

Analysis followed a predefined plan, used STATA V.14.1.1, reported using CONSORT guidelines.^30^ Analyses were conducted on an intention-to-treat (ITT) basis without imputation of missing data, adjusting for baseline value and centre. Primary effectiveness analysis used linear regression to estimate an adjusted mean difference comparing fatigue impact at 26 weeks (primary outcome) between groups as randomised. Analysis was repeated including 20 sets of imputed missing primary outcome data. The multiple imputation model included all variables that were part of the ITT primary analysis, variables potentially related to fatigue impact (fatigue severity, pain, self-rated and nurse-rated disease activity) and baseline variables that were associated with missingness of the primary outcome. Secondary analysis compared groups across 26, 52, 78 and 104 weeks using a mixed effects model (treatment arm baseline measure of the outcome, centre and timepoint as fixed effects and participant as a random effect to account for the repeated measures nature of the data). Further secondary analyses of the primary outcome included: restricting the analysis to baseline-eligible participants (ie, fatigue severity >6/10); a Complier Average Causal Effect (CACE) analysis to investigate the efficacy of the intervention (based on treatment compliance status) for comparison with the ITT estimate of the offer of the intervention^31^ and investigation of potential clustering by centre and group. Secondary outcomes at 26 weeks were examined using linear regression for continuous outcomes and logistic regression for binary outcomes (adjusted for baseline measure of the outcome and centre) and over 26, 52, 78 and 104 weeks using the mixed effects models above. Potential predictors of fatigue impact change were modelled using stepwise deletion. Exploratory analyses assessed the effects of a potential tutor learning curve, the week 14 consolidation session and individual self-efficacy (RASE) questions at 26 weeks (t-test).

### Patient involvement

Patient partners (Rooke, Robinson) brought experiential knowledge of RA fatigue and received the original CBT intervention.^11^ They advised on outcomes, questionnaires, handouts, recruitment and supported tutor training; Rooke provided advice on the tutor manual, attended project meetings and interpreted results.

## Results

**Figure 1:**
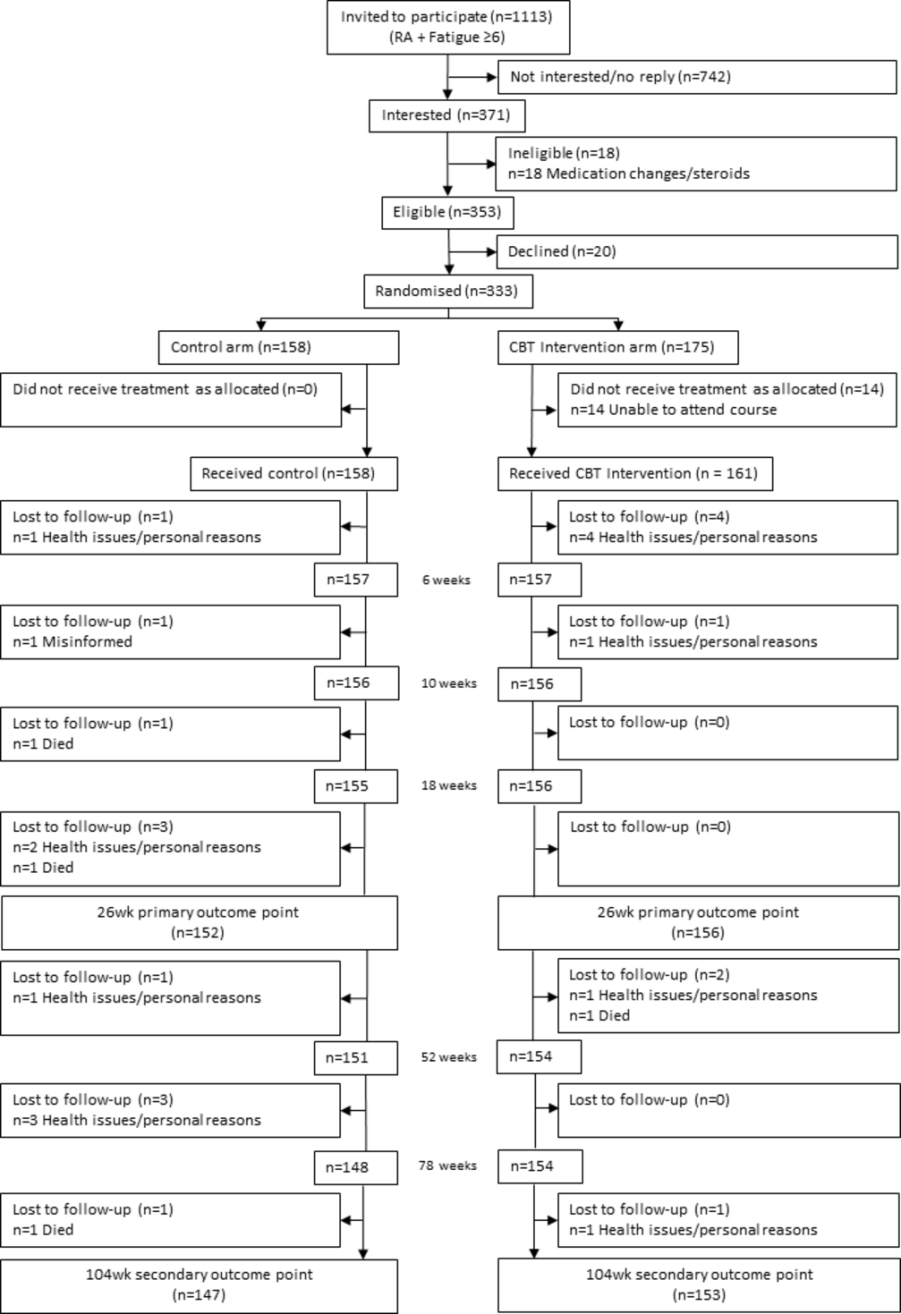
Participant flowchart. CBT, cognitive behavioural therapy.

Four consecutive cohorts of 9–16 patients were randomised in each of 7 centres over 2 years (n=333) (figure 1), with similar numbers across centres (see online supplementary data). Six of 158 patients randomised to usual care alone withdrew before week 26. While 6 of 175 patients randomised to RAFT attended a later course than planned (with fresh baseline data), 14 patients could not meet any local course dates and did not receive RAFT. Of the 161 patients who received RAFT, 4 withdrew for personal/health reasons (having attended 1–3 of the 7 sessions) and 1 before week 10. Therefore 156/175 RAFT patients (89.9%) and 152/158 control patients (96.0%) provided primary week 26 outcome data; 153/175 RAFT and 147/158 control patients provided week 104 data (87.4%, 93.0%).

### RAFT delivery

All 7 rheumatology centres provided at least two clinicians and of 15 trained, 14 went on to deliver RAFT: 4 nurse/occupational therapist pairs, 1 occupational therapist pair and 2 nurse pairs. Tutors had been qualified for a mean 18 years (6–30), with 5.3 years rheumatology experience (0–17); 10 had experience of group work, of whom 4 reported knowledge of goal-setting or CBT. All hospitals delivered four RAFT courses. Tutor pairs remained unchanged, apart from one centre where tutor absence was covered by the remaining tutor codelivering one course with a trainer and one with a colleague who had previously observed. Seven of the 196 RAFT sessions (28 courses of 7 sessions) were delivered by a single tutor due to absence (3.6%). Independent monitoring of two sessions of every course confirmed tutor fidelity to RAFT content, delivery and CB principles.

### RAFT attendance

At randomisation, RAFT groups averaged six patients (5-8). Patients attended a mean 5.85 of their 7 RAFT sessions (SD 1.63), with 136 (87.2%) attending 5–7. All 156 RAFT patients attended their Session 1 (definition of having received RAFT), and each of the individual Sessions 2–7 was attended by >75% randomised patients. No related adverse events reported.

### Baseline data

Total 308/333 randomised patients completed primary 26 week outcome data. Baseline characteristics were similar between arms, being primarily female (RAFT 125/156, 80.1%, control 121/152, 79.6%), >60 years old (RAFT median 63.7, IQR 54.2, 69.9; control 61.8, IQR 54.4, 69.6) and a median 10 year disease duration (RAFT IQR 5, 19; control 3, 20). Baseline clinical data were similar between arms (table 1) with high fatigue impact (mean BRAF-NRS Effect >7/10) that was slightly higher than fatigue severity, low perceived ability to cope with fatigue, relatively high baseline disease activity (mean DAS28>4.2), moderate pain, low disability, moderate self-efficacy and helplessness. The 25 patients who withdrew had similar characteristics (see online supplementary data). During the trial, normal clinical management meant patients had changes to major RA medications but this was not different between arms (see online supplementary data).

**Table 1:** Baseline clinical data

	Control (n=158)		RAFT (n=175)	
	n (%)*	Mean (SD)	n (%)*	Mean (SD)
Fatigue				
BRAF-NRS Effect (0–10)	152 (96.2)	7.23 (1.6)	156 (89.1)	7.10 (1.7)
BRAF-NRS Severity (0–10)	142 (89.9)	6.85 (1.57)	152 (86.9)	6.89 (1.57)
BRAF-NRS Coping (0–10)†	142 (89.9)	4.84 (2.09)	152 (86.9)	5.16 (2.08)
BRAF-MDQ impact overall (0–70)	142 (89.9)	40.39 (12.99)	152 (86.9)	40.42 (12.70)
BRAF-MDQ Physical (0–22)	142 (89.9)	16.19 (3.21)	152 (86.9)	16.12 (3.39)
BRAF-MDQ Emotional (0–12)	142 (89.9)	6.71 (3.31)	152 (86.9)	6.55 (3.18)
BRAF-MDQ Cognitive (0–15)	142 (89.9)	7.58 (4.04)	152 (86.9)	7.54 (4.00)
BRAF-MDQ Living with (0–21)	142 (89.9)	9.90 (5.18)	152 (86.9)	10.21 (5.05)
Pain NRS (0–10)	142 (89.9)	5.57 (2.10)	152 (86.9)	5.70 (2.12)
Disability MHAQ (0–3)	142 (89.9)	0.76 (0.51)	151 (86.3)	0.75 (0.53)
Quality of life AIMS VAS (0–100)	141 (89.2)	49.89 (20.44)	152 (86.9)	49.16 (22.27)
Disease activity				
Assessed—DAS28 (0.96+)	145 (91.8)	4.23 (1.11)	147 (84.0)	4.22 (1.30)
Self-report—sPDAS2 (2.4–7.9)	142 (89.9)	4.36 (0.99)	151 (86.3)	4.44 (1.06)
Anxiety HADS (0–21)	142 (89.9)	8.01 (4.45)	151 (86.3)	7.29 (4.08)
Depression HADS (0–21)	142 (89.9)	6.79 (3.94)	151 (86.3)	7.18 (3.59)
Valued life activities (0–3)	142 (89.9)	1.08 (0.60)	151 (86.3)	1.16 (0.61)
Helplessness AHI (5-30)	142 (89.9)	18.98 (4.74)	152 (86.9)	19.03 (4.67)
Self-efficacy RASE (28-140)††	142 (89.9)	104.38 (11.34)	151 (86.3)	102.49 (11.51)
Sleep quality	142 (89.9)		149 (85.1)	
Very good‡		5 (3.5%)		9 (6.0%)
Fairly good‡		58 (40.9%)		48 (32.2%)
Fairly bad‡		51 (35.9%)		56 (37.6%)
Very bad‡		28 (19.7%)		36 (24.2%)

*Percentage of total randomised (Control 158, RAFT 175).

†Higher score=better outcome.

‡Percentage of questionnaires returned.

AHI, Arthritis Helplessness Index; AIMS, Arthritis Impact Measurement Scale; BRAF-MDQ, Bristol RA Fatigue Multidimensional Questionnaire; BRAF-NRS, Bristol RA Fatigue Numerical Rating Scale; HADS, Hospital Anxiety and Depression Scale; MHAQ, Modified Health Assessment Questionnaire; RASE, RA Self-Efficacy.

### Primary outcome

At week 26, mean BRAF-NRS impact was reduced by 1.36 units (p<0.001) in RAFT compared with 0.88 in controls (p<0.004).^32 33^ Regression analysis showed a difference between changes in fatigue impact NRS of −0.59 in favour of RAFT (p=0.03, table 2), giving a standardised effect size of 0.36 (adjusted difference in mean/pooled baseline SD).

**Table 2:** Primary outcome of fatigue impact at 26 weeks

	Control			RAFT			Adjusted* mean difference (95% CI)	P value
	n (%)†	Week 0 Mean (SD)	Week 26 Mean (SD)	n (%)†	Week 0 Mean (SD)	Week 26 Mean (SD)		
BRAF-NRS effect	152 (96.2)	7.23 (1.6)	6.36 (2.42)	156 (89.1)	7.10 (1.7)	5.74 (2.41)	−0.59 (−1.11 to -0.06)	0.03

*Linear regression adjusted for baseline BRAF-NRS impact and centre.

†Percentage of total randomised (Control 158, RAFT 175).

BRAF-NRS, Bristol RA Fatigue Numerical Rating Scale.

The primary analysis was repeated excluding patients who had fallen below the BRAF-NRS Severity>6/10 eligibility criterion between screening and baseline (23/156 RAFT, 23/152 control). For baseline-eligible patients, a larger effect was seen in favour of RAFT, with an adjusted mean difference between arms for fatigue impact of −0.82 (95% CI −1.40 to -0.24, p=0.01).

Analysis on 20 sets of imputed data gave almost identical results (adjusted mean difference between arms for fatigue impact −0.58), therefore no further imputation was used. CACE analysis supported the finding of a larger effect from RAFT in those who ‘complied’ with the intervention compared with the ITT estimate of the ‘offer’ of the intervention (CACE treatment effect estimate −0.69). However, CACE methods could be deemed inappropriate as 14 patients randomised to but not attending RAFT had no follow-up data (see online supplementary data). No evidence of clustering was demonstrated: log-likelihood of linear mixed model −696.44 vs log-likelihood of model not adjusting for clustering −696.48 (p=0.96).

### Primary outcome over two years

**Figure 2:**
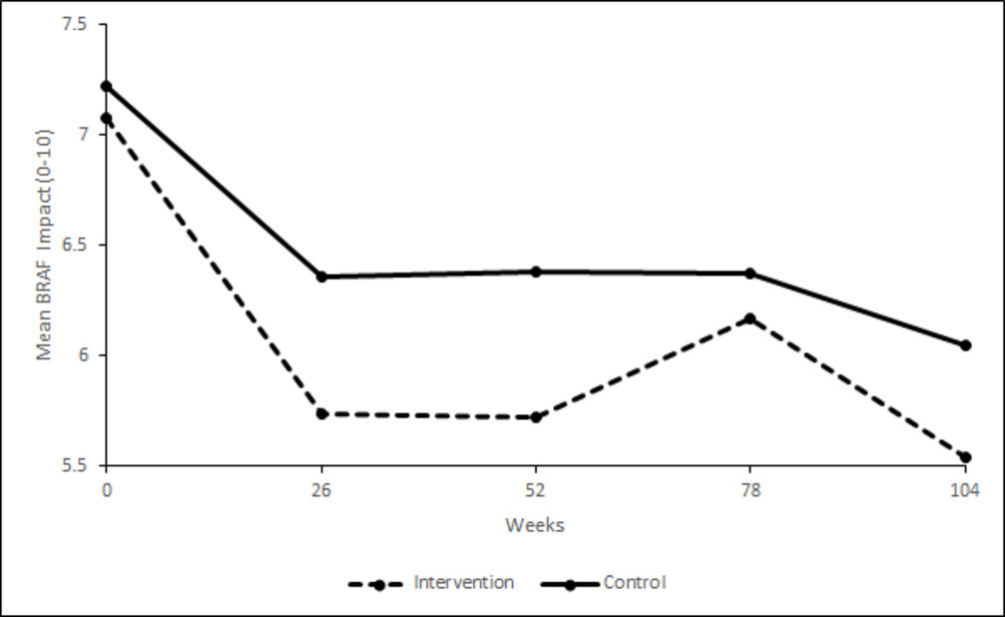
BRAF-NRS Impact scores (0–10) over weeks 0–104 by trial arm. BRAF-NRS, Bristol RA Fatigue Numerical Rating Scale.

Difference between the arms for fatigue impact was maintained over 2 years (figure 2). RAFT patients had a BRAF-NRS Impact score that was on average better than controls over 2 years with an adjusted mean difference of −0.49 (95% CI −0.83 to -0.14, p=0.01) (see online supplementary data). The mixed effects model conducted on baseline-eligible patients again showed a slightly larger treatment effect with an average adjusted mean difference over 2 years of −0.58 (95% CI −0.95 to -0.22, p=0.002).

### Secondary outcomes

There was a difference between arms at 26 weeks in favour of RAFT for overall fatigue impact, emotional fatigue, living with fatigue (BRAF-MDQ Overall and subscales) and self-efficacy (RASE) (table 3). Findings were similar over 2 years, when better outcomes for fatigue coping (BRAF-NRS Coping) also emerged for RAFT whereas self-efficacy was no longer significant (table 4). There was no difference between arms for the other 13 secondary outcomes, including fatigue severity (tables 3 and 4).

**Table 3:** Adjusted mean difference between arms in secondary outcomes at 26 weeks

	Control		RAFT		Adjusted mean difference* (95% CI)	P value	Effect size
	n (%)†	Mean (SD)	n (%)†	Mean (SD)			
Fatigue							
BRAF-NRS Severity (0–10)	142 (89.9)	6.13 (2.30)	152 (86.9)	5.91 (2.22)	−0.24 (−0.75 to 0.27)	0.35	
BRAF-NRS Coping (0-10)‡	142 (89.9)	5.32 (2.42)	152 (86.9)	5.25 (2.33)	−0.15 (−0.69 to 0.39)	0.58	
BRAF-MDQ Impact Overall (0–70)	142 (89.9)	34.74 (16.41)	152 (86.9)	31.51 (16.02)	−3.42 (−6.44 to -0.39)	0.03	0.27
BRAF-MDQ Physical (0–22)	142 (89.9)	14.40 (5.23)	152 (86.9)	13.72 (4.91)	−0.68 (−1.78 to 0.42)	0.23	
BRAF-MDQ Emotional (0–12)	142 (89.9)	5.36 (3.79)	152 (86.9)	4.37 (3.51)	−0.91 (−1.58 to -0.23)	0.01	0.28
BRAF-MDQ Cognitive (0–15)	142 (89.9)	6.55 (4.16)	152 (86.9)	5.89 (4.35)	−0.66 (−1.45 to 0.13)	0.10	
BRAF-MDQ Living with (0–21)	142 (89.9)	8.43 (5.68)	152 (86.9)	7.53 (5.43)	−1.19 (−2.17 to -0.21)	0.02	0.23
Pain NRS (0–10)	142 (89.9)	5.24 (2.41)	152 (86.9)	5.47 (2.32)	0.16 (−0.33 to 0.65)	0.51	
Disability MHAQ (0–3)	142 (89.9)	0.70 (0.51)	151 (86.3)	0.71 (0.54)	0.02 (−0.06 to 0.10)	0.67	
Quality of Life AIMS VAS (0–100)	141 (89.2)	47.70 (23.04)	152 (86.9)	47.22 (23.46)	−0.33 (−5.13 to 4.65)	0.90	
Disease Activity:							
Assessed—DAS28 (0.96+)	145 (91.8)	4.10 (1.31)	147 (84.0)	4.13 (1.38)	0.02 (−0.21 to 0.24)	0.88	
Self-report—sPDAS2 (2.4–7.9)	142 (89.9)	4.33 (1.04)	151 (86.3)	4.44 (1.13)	0.05 (−0.16 to 0.26)	0.63	
Anxiety HADS (0–21)	142 (89.9)	7.56 (4.48)	151 (86.3)	6.65 (4.36)	−0.33 (−0.95 to 0.29)	0.30	
Depression HADS (0–21)	142 (89.9)	6.42 (4.06)	151 (86.3)	6.22 (3.76)	−0.50 (−1.14 to 0.14)	0.13	
Valued Life Activities (0–3)	142 (89.9)	1.07 (0.62)	151 (86.3)	1.09 (0.67)	−0.05 (−0.15 to 0.05)	0.34	
Helplessness AHI (5-30)	142 (89.9)	17.47 (5.46)	152 (86.9)	16.92 (5.06)	−0.61 (−1.65 to 0.43)	0.25	
Self-efficacy RASE (28-140)‡	142 (89.9)	104.67 (13.31)	151 (86.3)	106.26 (14.78)	3.05 (0.43 to 5.66)	0.02	0.27
Sleep quality:							
Very good§		9 (6.3%)		17 (11.4%)	0.75 (0.47 to 1.17)¶	0.21	
Fairly good§		65 (45.8%)		64 (43.0%)			
Fairly bad§		51 (35.9%)		51 (34.2%)			
Very bad§		17 (12.0%)		17 (11.4%)			

*Linear regression adjusted for baseline measure of outcome and centre.

†Percentage of total randomised (Control 158, RAFT 175).

‡Higher score=better outcome.

§Number of patients (% questionnaires returned).

¶OR from ordinal logistic regression.

AHI, Arthritis Helplessness Index; AIMS, Arthritis Impact Measurement Scale; BRAF-MDQ, Bristol RA Fatigue Multidimensional Questionnaire; BRAF-NRS, Bristol RA Fatigue Numerical Rating Scale; HADS, Hospital Anxiety and Depression Scale; MHAQ, Modified Health Assessment Questionnaire; RASE, RA Self-Efficacy.

**Table 4:** Mixed effects models examining effect of raft on secondary outcomes over 2 years

	Adjusted mean difference*	95% CI	P value
Fatigue			
BRAF-NRS Severity (0–10)	−0.17	−0.54 to 0.20	0.38
BRAF-NRS Coping (0–10)†	0.42	0.08 to 0.77	0.02
BRAF-MDQ Impact Overall (0–70)	−2.98	−5.39 to -0.57	0.02
BRAF-MDQ Physical (0–22)	−0.64	−1.45 to 0.17	0.12
BRAF-MDQ Emotional (0–12)	−0.90	−1.44 to -0.37	0.001
BRAF-MDQ Cognitive (0–15)	−0.53	−1.14 to 0.08	0.09
BRAF-MDQ Living with (0–21)	−0.93	-1.75 to -0.10	0.03
Pain NRS (0–10)	0.01	−0.38 to 0.40	0.94
Disability MHAQ (0–3)	0.01	−0.07 to 0.08	0.86
Quality of Life AIMS VAS (0–100)	−0.02	−3.91 to 3.86	0.99
Disease activity sPDAS2 (2.4–7.9)	0.03	−0.15 to 0.20	0.77
Anxiety HADS (0–21)	−0.40	−0.96 to 0.15	0.16
Depression HADS (0–21)	−0.49	−1.06 to 0.08	0.09
Valued Life Activities (0–3)	−0.06	−0.14 to 0.03	0.22
Helplessness AHI (5-30)	−0.27	−1.12 to 0.58	0.54
Self-efficacy RASE (28-140)†	1.31	−0.80 to 3.42	0.23
Sleep Quality	0.74‡	0.44 to 1.27	0.28

*Adjusted for baseline measure of outcome and centre.

†Higher score=better outcome (for all other scales lower score=better outcome).

‡OR from ordinal logistic regression.

AHI, Arthritis Helplessness Index; AIMS, Arthritis Impact Measurement Scale; BRAF-MDQ, Bristol RA Fatigue Multidimensional Questionnaire; BRAF-NRS, Bristol RA Fatigue Numerical Rating Scale; HADS, Hospital Anxiety and Depression Scale; MHAQ, Modified Health Assessment Questionnaire; RASE, RA Self-Efficacy.

### Patient satisfaction

At 26 weeks, 89% of RAFT patients (133/150) rated course satisfaction >8/10 compared with 54% (75/139) controls rating booklet satisfaction (p<0.0001). Additionally, 96% of RAFT patients (144/150) were very likely to recommend it to others (scoring >8/10) compared with 68% (95/139) controls recommending the booklet (p<0.001). At 52 weeks, 20% of RAFT patients (n=35) had made further contact with their RAFT group, and 9% (16) at 104 weeks.

### Potential predictors of fatigue impact change

The final linear regression model suggested worse outcome at 26 weeks was predicted by being female (0.75 unit increase in BRAF-NRS Effect, 95% CI 0.08 to 1.41, p=0.03) and higher baseline disease activity (1 unit increase in baseline DAS28 associated with 0.25 unit increase in BRAF-NRS Effect, 95% CI 0.02 to 0.49, p=0.04).

*Exploratory analyses* (see online supplementary data): The difference between arms in mean fatigue impact change was smallest in cohort 1 (−0.37 BRAF-NRS Effect) and greatest in cohort 4 (−0.82), suggesting possible tutor learning over time (the study was not powered to test this). The adjusted mean differences between arms for fatigue impact, coping with fatigue and emotional fatigue were greater at week 18 than week 10, suggesting benefit from the week 14 consolidation session, but could reflect random variation over time. RASE addresses self-efficacy for RA self-management, and six RASE items demonstrated a difference between arms for change in favour of RAFT at week 26: relaxation, pacing, accepting fatigue, asking for help, using relaxation tapes and avoiding things that cause pain.

## Discussion

RAFT, a seven-session group CBA course for RA fatigue self-management delivered by rheumatology nurses and occupational therapists, reduced fatigue impact beyond usual care both at 6 months and 2 years. RAFT had high patient attendance and satisfaction. Improvements were also seen in emotional fatigue, living with fatigue, coping with fatigue and self-efficacy.

Improvements in fatigue impact demonstrated in usual care alone were not seen in the previous RCT, where the control was one general self-management group session of didactic information.^11^ The usual care fatigue booklet^18^ in this RCT was written by the team following the original RCT.^12^ A qualitative study suggested that patients felt the booklet made them feel more responsible for taking actions to manage their fatigue.^34^ The brief booklet discussion with the nurse in this RCT may have had a greater effect than a patient picking up a booklet in clinic.

The amount of fatigue impact change that might be clinically important for people with RA is unknown. The minimal clinically important difference (MCID) for RA fatigue severity scales averages 10%,^35–37^ while multiple sclerosis fatigue impact MCID is 15%.^38 39^ RAFTs demonstrated 19% change in fatigue impact and usual care 12% (–1.36, –0.88 units) are thus probably clinically meaningful.

There was no treatment difference between arms for fatigue severity. Longitudinal studies demonstrate RA fatigue severity is largely stable with most having a persistent moderately high/high trajectory.^5 40^ Symptom severity differs from impact: patients can report low impact from severe symptoms and vice versa.^41 42^ Impact may be the product of interactions between symptom severity, personal importance and perceived coping.^43^ The 2 year treatment difference in favour of RAFT for coping with fatigue might reflect reduced personal importance of fatigue, thus improving fatigue impact even with persistent fatigue severity. RAFT participants may have made a shift in how they view and manage fatigue, finding ways to reduce its impact.^44^

The 2-year effects of RAFT on fatigue coping suggest that patients’ newly acquired fatigue self-management skills became embedded. Core self-management skills taught in CB approaches may have translated into improved coping. Overall self-efficacy favoured RAFT at 26 weeks with changes in 6/28 RASE items of key fatigue self-management issues addressed in RAFT. Self-efficacy was no longer significant at 2 years, possibly reflecting the dominance of the 22 general RA self-management items in the RASE score over time since RAFT, compared with the focused fatigue coping NRS (ie, a measurement issue).

### Strengths and limitations

The study’s external validity was strengthened by involving 7 hospitals, 14 tutors, broad entry criteria and strong patient and public involvement. The pragmatic trial design accommodated features of real clinical practice: natural variations in medication, fatigue, group size, session attendance and tutor availability. Collection of the primary outcome by telephone gave high return rates.

There was no control for social effects of RAFT groups: seven sessions of didactic information would not reflect usual care and likely incur high attrition. In the original RCT, patients reported benefit from interactions with other patients, but considered tutors more important, otherwise patients would have ‘pulled one another down’.^44^ Delay between screening and baseline assessment was inevitable while building cohorts for randomisation, and some patients no longer had eligible fatigue severity scores at baseline. However, RAFT still had an overall treatment effect, with a greater effect in baseline-eligible participants. Delays would be shorter in clinical practice as only sufficient patients for a RAFT group need be recruited. Six RAFT patients requested a later course and had a fresh baseline assessment, collected after randomisation. Major medication changes might influence fatigue but changes were not different between arms and remission through optimal medication does not resolve problematic fatigue.^45^ We did not collect follow-up data on 25 patients who withdrew, but imputing missing data did not change the primary outcome analysis. RAFT patients were not asked to rate the usual care booklet.

### Implications for research

Codelivery by a rheumatology professional and a patient (bringing experiential knowledge of RA fatigue) could be evaluated,^46^ as could delivery in physical long-term conditions with fatigue (multiple sclerosis, Parkinson’s disease) and the MCID for the BRAF-NRS Effect established.

### Implications for healthcare

The CBA intervention was delivered by clinical rheumatology nurses and occupational therapists, addressing the lack of rheumatology psychologists.^12^ Similar success has been demonstrated in multiple sclerosis fatigue.^47^ RAFT delivery by clinical teams was feasible: all planned RAFT courses were delivered, tutor absence was managed and patient attendance high. Increasing group size to 8–10 patients would be feasible, as would including patients with other inflammatory rheumatic diseases and fatigue.^48 49^ Several RCT centres now deliver RAFT clinically.

## References

[1] Walter MJM, Kuijper TM, Hazes JMW, et al Fatigue in early, intensively treated and tight-controlled rheumatoid arthritis patients is frequent and persistent: a prospective study. Rheumatol Int 2018;38:1643–50. 10.1007/s00296-018-4102-5 30014260 PMC6105154

[2] Hewlett S, Cockshott Z, Byron M, et al Patients' perceptions of fatigue in rheumatoid arthritis: overwhelming, uncontrollable, ignored. Arthritis Rheum 2005;53:697–702. 10.1002/art.21450 16208668

[3] Repping Wuts H, Uitterhoeve R, van Riel P, et al Fatigue as experienced by patients with rheumatoid arthritis. Int J Nurs Stud 2008;45:995–1002. 17662291 10.1016/j.ijnurstu.2007.06.007

[4] Druce KL, Jones GT, Macfarlane GJ, et al The longitudinal course of fatigue in rheumatoid arthritis: results from the norfolk arthritis register. J Rheumatol 2015;42:2059–65. 10.3899/jrheum.141498 26472420

[5] Repping-Wuts H, Hewlett S, van Riel P, et al Fatigue in patients with rheumatoid arthritis: British and Dutch nurses’ knowledge, attitudes and management. J Adv Nursing 2009;65:901–11. 10.1111/j.1365-2648.2008.04904.x 19243466

[6] Nikolaus S, Bode C, Taal E, et al Fatigue and factors related to fatigue in rheumatoid arthritis: a systematic review. Arthritis Care Res 2013;65:1128–46. 10.1002/acr.21949 23335492

[7] Hewlett S, Chalder T, Choy E, et al Fatigue in rheumatoid arthritis: time for a conceptual model. Rheumatology 2011;50:1004–6. 10.1093/rheumatology/keq282 20819797

[8] Almeida C, Choy EHS, Hewlett S, 2016 Biologic interventions for fatigue in rheumatoid arthritis (systematic review). Cochrane systematic reviews. Available: https://www.cochranelibrary.com/cdsr/ [Accessed 5 Sep 2018]. 10.1002/14651858.CD008334.pub2 PMC7175833 27271314

[9] Cramp F, Hewlett S, Almeida C, et al Non-pharmacological interventions for fatigue in rheumatoid arthritis. Cochrane Database of Systematic Reviews 2013;44:CD008322 10.1002/14651858.CD008322.pub2 PMC11748118 23975674

[10] Evers AWM, Kraaimaat FW, van Riel PLCM, et al Tailored cognitive-behavioral therapy in early rheumatoid arthritis for patients at risk: a randomized controlled trial. Pain 2002;100:141–53. 10.1016/S0304-3959(02)00274-9 12435467

[11] Hewlett S, Ambler N, Almeida C, et al Self-management of fatigue in rheumatoid arthritis: a randomised controlled trial of group cognitive-behavioural therapy. Ann Rheum Dis 2011;70:1060–7. 10.1136/ard.2010.144691 21540202 PMC3086034

[12] Dures E, Almeida C, Caesley J, et al A survey of psychological support provision for people with inflammatory arthritis in secondary care in England. Musculoskelet Care 2014;12:173–81. 10.1002/msc.1071 PMC4282402 24753071

[13] Hewlett S, Ambler N, Almeida C, et al Protocol for a randomised controlled trial for reducing arthritis fatigue by clinical teams (raft) using cognitive-behavioural approaches. BMJ Open 2015;5:e009061 10.1136/bmjopen-2015-009061 PMC4538284 26251413

[14] Arnett FC, Edworthy SM, Bloch DA, et al The American rheumatism association 1987 revised criteria for the classification of rheumatoid arthritis. Arthritis Rheum 1988;31:315–24. 10.1002/art.1780310302 3358796

[15] Nicklin J, Cramp F, Kirwan J, et al Measuring fatigue in rheumatoid arthritis: a cross-sectional study to evaluate the Bristol rheumatoid arthritis fatigue multi-dimensional questionnaire, visual analog scales, and numerical rating scales. Arthritis Care Res 2010;62:1559–68. 10.1002/acr.20282 20583112

[16] Sage N, Sowden M, Chorlton E Guided discovery: using the Socratic method : Sage N, Sowden M, Chorlton E, Cognitive Behaviour Therapy for Chronic Illness and Palliative Care. Chichester: Wiley, 2008: 66–77.

[17] Bandura A Self-efficacy: toward a unifying theory of behavioral change. Psychol Rev 1977;84:191–215. 10.1037/0033-295X.84.2.191 847061

[18] Hewlett S, Ambler N, Dures E, 2011 Arthritis Research UK. self-help and daily living: fatigue and arthritis. Available: www.arthritisresearchuk.org/~/media/Files/Arthritis-information/Living-with-arthritis/2269-Fatigue-and-Arthritis-inc-excercise-book.ashx [Accessed 11 May 2018].

[19] Pincus T, Summey JA, Soraci SA, et al Assessment of patient satisfaction in activities of daily living using a modified Stanford health assessment Questionnaire. Arthritis Rheum 1983;26:1346–53. 10.1002/art.1780261107 6639693

[20] Buysse DJ, Reynolds CF, Monk TH, et al The Pittsburgh sleep quality index. Psychiatry Res 1989;28:193–213. 2748771 10.1016/0165-1781(89)90047-4

[21] van der Heijde DM, van ‘t Hof M, van Riel PL, et al Development of a disease activity score based on judgement in clinical practice by rheumatologists. J Rheumatol 1993;20:579–81. 8478878

[22] Choy EH, Khoshaba B, Cooper D, et al Development and validation of a patient-based disease activity score in rheumatoid arthritis that can be used in clinical trials and routine practice. Arthritis Rheum 2008;59:192–9. 10.1002/art.23342 18240256

[23] Choy E, Leung AMH THU0083 Simplified Patient-Derived Disease Activity Score (SPDAS2): A Simplified Version without Early Morning Stiffness. Ann Rheum Dis 2016;75(Suppl 2):209.1–209. 10.1136/annrheumdis-2016-eular.1443

[24] Zigmond AS, Snaith RP The hospital anxiety and depression scale. Acta Psychiatr Scand 1983;67:361–70. 10.1111/j.1600-0447.1983.tb09716.x 6880820

[25] Meenan RF, Gertman PM, Mason JH Measuring health status in arthritis: the arthritis impact measurement scales. Arthritis Rheum 1980;23:2:146–52. 7362665 10.1002/art.1780230203

[26] Katz PP, Morris A, Yelin EH Prevalence and predictors of disability in valued life activities among individuals with rheumatoid arthritis. Ann Rheum Dis 2006;65:763–9. 10.1136/ard.2005.044677 16249225 PMC1798183

[27] Hewlett S et al Development and validation of a self-efficacy scale for use in British patients with rheumatoid arthritis (RASE). Rheumatology 2001;40:1221–30. 10.1093/rheumatology/40.11.1221 11709605

[28] Stein MJ, Wallston KA, Nicassio PM Factor structure of arthritis helplessness index. J Rheumatol 1988;15:427–32. 3379620

[29] Lamb SE, Hansen Z, Lall R, et al Group cognitive behavioural treatment for low-back pain in primary care: a randomised controlled trial and cost-effectiveness analysis. Lancet 2010;375:916–23. 10.1016/S0140-6736(09)62164-4 20189241

[30] CONSORT, 2018 (consolidating standards of reporting trials) statement, checklist and flowchart. Available: www.consort-statement.org [Accessed 10 Apr 2018].

[31] Connell AM Employing complier average causal effect analytic methods to examine effects of randomized encouragement trials. Am J Drug Alcohol Abuse 2009;35:253–9. 10.1080/00952990903005882 20180678 PMC2845534

[32] Hewlett S, Ambler N, Almeida C Reducing arthritis fatigue – clinical teams (raft) using cognitive behavioural approaches: an RCT. Ann Rheum Dis 2017;76110. 10.1136/annrheumdis-2018-214469 PMC6530078 30793700

[33] Hewlett S, Ambler N, Almeida C Reducing arthritis fatigue – clinical teams (raft) using cognitive behavioural approaches: an RCT. Rheumatology 2018;57:key075.180. 10.1136/bmjopen-2015-009061 PMC4538284 26251413

[34] Hart RI, Ng W-F, Newton JL, et al What impact does written information about fatigue have on patients with autoimmune rheumatic diseases? Findings from a qualitative study. Musculoskelet Care 2017;15:230–7. 10.1002/msc.1164 PMC5600097 27860255

[35] Khanna D, Pope JE, Khanna PP, et al The minimally important difference for the fatigue visual analog scale in patients with rheumatoid arthritis followed in an academic clinical practice. J Rheumatol 2008;35:2339–43. 10.3899/jrheum.080375 19004044 PMC3155760

[36] Wells G, Li T, Maxwell L, et al Determining the minimal clinically important differences in activity, fatigue, and sleep quality in patients with rheumatoid arthritis. J Rheumatol 2007;34:280–9. 17304654

[37] Kherani RB J, Brant R, Lacaille D, et al Determination of the minimal clinically important difference for seven fatigue measures in rheumatoid arthritis. J Clin Epidemiol 2008;61:705–13. 18359189 10.1016/j.jclinepi.2007.08.016 PMC2486378

[38] Learmonth YC, Dlugonski D, Pilutti LA, et al Psychometric properties of the fatigue Severity Scale and the modified fatigue Impact Scale. J Neurol Sci 2013;331:102–7. 10.1016/j.jns.2013.05.023 23791482

[39] Kos D, Duportail M, D'hooghe M, et al Multidisciplinary fatigue management programme in multiple sclerosis: a randomized clinical trial. Mult Scler 2007;13:996–1003. 10.1177/1352458507078392 17623738

[40] van Steenbergen HW, Tsonaka R, Huizinga TWJ, et al Fatigue in rheumatoid arthritis; a persistent problem: a large longitudinal study. RMD Open 2015;1:e00041:e000041 10.1136/rmdopen-2014-000041 PMC4612698 26509063

[41] Hewlett S, Smith AP, Kirwan JR Measuring the meaning of disability in rheumatoid arthritis: the personal impact health assessment Questionnaire (PI HAQ). Ann Rheum Dis 2002;61:986–93. 10.1136/ard.61.11.986 12379521 PMC1753935

[42] Seror R, Tubach F, Baron G, et al Measure of function in rheumatoid arthritis: individualised or classical scales? Ann Rheum Dis 2010;69:97–101. 10.1136/ard.2008.102137 19346220 PMC2919538

[43] Sanderson TC, Hewlett SE, Flurey C, et al The impact triad (severity, importance, self-management) as a method of enhancing measurement of personal life impact of rheumatic diseases. J Rheumatol 2011;38:191–4. 10.3899/jrheum.100700 21285178

[44] Dures E, Kitchen K, Almeida C, et al “They didn't tell us, they made us work it out ourselves”: Patient perspectives of a cognitive-behavioral program for rheumatoid arthritis fatigue. Arthritis Care Res 2012;64:494–501. 10.1002/acr.21562 22162339

[45] Druce KL, Bhattacharya Y, Jones GT, et al Most patients who reach disease remission following anti-TNF therapy continue to report fatigue: results from the british Society for rheumatology biologics register for rheumatoid arthritis. Rheumatology 2016;55:1786–90. 10.1093/rheumatology/kew241 27330158

[46] Turner A, Anderson JK, Wallace LM, et al An evaluation of a self-management program for patients with long-term conditions. Patient Educ Counsel 2015;98:213–9. 10.1016/j.pec.2014.08.022 25441096

[47] Thomas S, Thomas PW, Kersten P, et al A pragmatic parallel arm multi-centre randomised controlled trial to assess the effectiveness and cost-effectiveness of a group-based fatigue management programme (facets) for people with multiple sclerosis. J Neurol Neurosurg Psychiatry 2013;84:1092–9. 10.1136/jnnp-2012-303816 23695501 PMC3786656

[48] Tench CM, McCurdie I, White PD, et al The prevalence and associations of fatigue in systemic lupus erythematosus. Rheumatology 2000;39:1249–54. 10.1093/rheumatology/39.11.1249 11085805

[49] Gudu T, Etcheto A, de Wit M, et al Fatigue in psoriatic arthritis – a cross-sectional study of 246 patients from 13 countries. Joint Bone Spine 2016;83:439–43. 10.1016/j.jbspin.2015.07.017 27055727

