# Supplementary material for: Reducing arthritis fatigue impact: two-year randomised controlled trial of cognitive behavioural approaches by rheumatology teams (RAFT)
Source: Ann Rheum Dis. 2019 Feb 6;78(4):465–72. doi: 10.1136/annrheumdis-2018-214469 (PMC6530078; doi:10.1136/annrheumdis-2018-214469)
Supplement: Supplementary data [file annrheumdis-2018-214469supp001.docx]

**RAFT RCT: Supplementary data file**

**Table A: RAFT session timetable**

**Table B: Randomisation by cohort and by centre (after Usual Care delivery)**

Table C: Baseline demographic data of patients who did/did not complete to 26 weeks

Table D: Baseline clinical data of patients who did not complete to 26 weeks

Table E: Patients making major medication changes during the trial

Table F: CACE analysis

Table G: Repeated measures analysis of BRAF-NRS Impact at 26, 52, 78 and 104 weeks

Table H: Change in BRAF-NRS Impact at 26 weeks for consecutive RAFT cohorts (1-4)

Table I Adjusted mean difference between arms for fatigue outcomes at 10 and 18

weeks (consolidation session occurred week 14)

Table J: RA Self-Efficacy (RASE) item changes 0-26 weeks (n=293)

**Table A: RAFT session timetable**

| **Wk** | **1^st^ hour** | **Supporting materials*** | **2^nd^ hour*** | | |
| --- | --- | --- | --- | --- | --- |
| 1 | Course purpose and expectations  Ground rules:  Commitment, confidentiality, homework  Validating fatigue: Share & discuss fatigue  experiences (difference from flare)  Self-management strategies, struggles and  difficulty of changing habits | H: Setting our course (groups’ ideas) | Energy management  -Boom & bust behaviour  -Rewards/pitfalls of this  -Prioritise, pace, plan,  -Choice is possible  H: Achieving balance  H: Activity cycling  T: Activity/rest diaries | | |
| 2 | What are your priorities for change, that  would 🡹QoL?  What are your drainers and energisers? | T: Wheel of life (priority areas) | Goal setting (two groups)  -Short/long-term goals  -Use peer group for ideas | | |
| 3 | Self-sabotage on the course  Sleep and rest:  Hours needed? Quality v quantity  Sleep hygiene strategies | H: Best ways of self-sabotage  H: Getting a better night’s sleep  T: Sleep diary (if needed) | Goal-setting review  Successes/barriers  New goals | | |
| 4 | Stress and relaxation  Personal stressors, bodily reactions  Relaxation rationale and techniques | H: Effects of stress  H: Relaxation practice guide  T: Relaxation CD | Goal-setting review  Successes/barriers  New goals | | |
| 5 | Assertiveness and communication  Passive, manipulative, assertive?  Other people’s reactions to these?  Communicating your needs | M: Cartoon examples  H: Saying ‘No’ | Goal-setting review  Successes/barriers  New goals | | |
| 6 | Review self-help tools  What have you learnt?  Review each topic  Dealing with setbacks – what could you do?  Negative self-talk, automatic thoughts,  rumination | M: Fatigue pit: Falling in/digging out  H: The pit  H: Coping with setbacks | Goal-setting review  Successes/barriers  New goals | | |
| 14 | Review last 8 wks;  Skills; dealing with setbacks;  New goals | M: Islands: Were on a Desert island (passive)  looking at the Mainland (100% health,ie  unrealistic). Now on Adaptive Coping  Island (realistic) | |  |  |

* H = Handouts, M = Metaphor, T = Tools

Reproduced from Hewlett S, Ambler N, Almeida C, Blair PS, Choy E, Dures E, Hammond A, Hollingworth W, Kirwan J, Plummer Z, Rooke C, Thorn J, Tomkinson K, Pollock J: Protocol for a randomised controlled trial for Reducing Arthritis Fatigue by clinical Teams (RAFT) using cognitive–behavioural approaches; BMJ Open 2015;5:e009061. doi:10.1136/bmjopen-2015-009061; an Open Access article distributed in accordance with the terms of the Creative Commons Attribution (CC BY 4.0) license, which permits others to distribute, remix, adapt and build upon the work, for commercial use, provided the original work is properly cited. See: <https://creativecommons.org/licenses/by-nc/4.0/>:

**Table B: Randomisation by cohort and by centre (after Usual Care delivery)**

**Cohort 1**

|  |  | **Allocation** | |
| --- | --- | --- | --- |
| **Centre** | **Randomised** | **Usual Care plus RAFT** | **Usual Care alone** |
| **1** | **10** | **5** | **5** |
| **2** | **13** | **7** | **6** |
| **3** | **16** | **8** | **8** |
| **4** | **11** | **6** | **5** |
| **5** | **15** | **8** | **7** |
| **6** | **12** | **6** | **6** |
| **7** | **12** | **6** | **6** |
| **Total** | **89** | **46** | **43** |

**Cohort 2**

|  |  | **Allocation** | |
| --- | --- | --- | --- |
| **Centre** | **Randomised** | **Usual Care plus RAFT** | **Usual Care alone** |
| **1** | **11** | **6** | **5** |
| **2** | **13** | **7** | **6** |
| **3** | **12** | **6** | **6** |
| **4** | **9** | **5** | **4** |
| **5** | **15** | **8** | **7** |
| **6** | **12** | **6** | **6** |
| **7** | **10** | **5** | **5** |
| **Total** | **82** | **43** | **39** |

**Cohort 3**

|  |  | **Allocation** | |
| --- | --- | --- | --- |
| **Centre** | **Randomised** | **Usual Care plus RAFT** | **Usual Care alone** |
| **1** | **12** | **6** | **6** |
| **2** | **9** | **5** | **4** |
| **3** | **12** | **6** | **6** |
| **4** | **15** | **8** | **7** |
| **5** | **15** | **8** | **7** |
| **6** | **13** | **7** | **6** |
| **7** | **9** | **5** | **7** |
| **Total** | **85** | **45** | **40** |

**Cohort 4**

|  |  | **Allocation** | |
| --- | --- | --- | --- |
| **Centre** | **Randomised** | **Usual Care plus RAFT** | **Usual Care alone** |
| **1** | **10** | **5** | **5** |
| **2** | **13** | **7** | **6** |
| **3** | **13** | **7** | **6** |
| **4** | **9** | **5** | **4** |
| **5** | **13** | **7** | **6** |
| **6** | **9** | **5** | **4** |
| **7** | **10** | **5** | **5** |
| **Total** | **77** | **41** | **36** |

**Table C: Baseline demographic data of patients who did/did not complete to 26 weeks**

|  | **Control**  **n=152** | **RAFT**  **n=156** | **Did not complete**  **n=25** |
| --- | --- | --- | --- |
| Female | 121 (79.6%) | 125 (80.1%) | 20 (80%) |
| Age (yrs)^a^ | 61.8 (54.4, 69.6) | 63.7 (54.2, 69.9) | 69 (61.3, 72) |
| Disease duration (yrs) ^a^ | 10 (3, 20) | 10 (5, 19) | 10 (5, 11) |
| Co-morbidity | 119 (78.8%) | 124 (80.0%) | 20 (80.0%) |
| Fatigue:  Severity (BRAF-NRS, 0-10)^b,c^ | 6.85 (1.57) | 6.89 (1.57) | 6.64 (1.47) |
| Impact (BRAF-NRS, 0-10)^b,c^ | 7.23 (1.6) | 7.10 (1.7) | 6.96 (1.54) |
| Other self-management course  Years since course ^a^ | 21 (14.0%)  8 (5, 11) | 16 (10.3%)  5 (3, 10) | 1 (4.0%)  2 |
| Socio-Economic status: |  |  |  |
| England: |  |  |  |
| Deprived | 28 (21.1%) | 23 (17.2%) | 6 (27.3%) |
| Moderate | 60 (45.1%) | 65 (48.5%) | 10 (45.5%) |
| Affluent | 45 (33.8%) | 46 (34.3%) | 6 (27.3%) |
| ….Wales: |  |  |  |
| Deprived | 7 (41.2%) | 7 (35.0%) | 2 (66.6%) |
| Moderate | 3 (17.7%) | 5 (25.0%) | 1 (33.3%) |
| Affluent | 7 (41.2%) | 8 (45.0%) | 0 |
| Ethnicity |  |  |  |
| White | 147 (98.0%) | 151 (96.8%) | 24 (96.0%) |
| Asian/Asian British | 3 (2.0%) | 5 (.01%) | 1 (4.0%) |

^a^Median (lower and upper quartile)

^b^High score is worse

^c^Mean (SD)

**Table D: Baseline clinical data of patients who did not complete to 26 weeks^a^**

|  | **Did not complete (n=25)** |
| --- | --- |
|  | **Mean (SD)** |
| Fatigue: |  |
| BRAF-NRS Impact (0-10) | 6.96 (1.5) |
| BRAF-NRS Severity (0-10) | 6.64 (1.5) |
| BRAF-NRS Coping (0-10)^b^ | 4.88 (2.0) |
| BRAF-MDQ Overall Impact (0-70) | 41.80 (11.8) |
| BRAF-MDQ Physical (0-22) | 15.52 (2.7) |
| BRAF-MDQ Emotional (0-12) | 7.28 (3.1) |
| BRAF-MDQ Cognitive (0-15) | 8.64 (3.2) |
| BRAF-MDQ Living with (0-21) | 10.36 (5.4) |
| Pain NRS (0-10) | 6.08 (2.3) |
| Disability MHAQ (0-3) | 0.87 (0.6) |
| Quality of Life AIMS VAS (0-100) | 52.40 (22.4) |
| Disease Activity:  Assessed - DAS28 (0.96+) | 4.25 (1.4) |
| Self-report - sPDAS2 (2.4-7.9) | 4.57 (1.0) |
| Anxiety HADs (0-21) | 8.88 (3.8) |
| Depression HADs (0-21) | 7.00 (3.9) |
| Valued Life Activities (0-3) | 1.17 (0.7) |
| Helplessness AHI (5-30) | 17.86 (5.7) |
| Self-efficacy RASE (28-140)^b^ | 101.19 (11.9) |
| Sleep quality^c^  Very good  Fairly good  Fairly bad  Very bad | 0 (0%)  11 (44%)  7 (28%)  7 (28%) |

^a^Similar to those who did complete (see *Table 5*)

^b^Higher score = better outcome

^c^Percentage of questionnaires returned

**Table E: Patients making major medication changes during the trial^a^**

|  | **n** | **Control**  **n (%)^b^** | **n** | **RAFT**  **n (%)^b^** | ***p*-value**  **(Pearson Chi-Squared test)** |
| --- | --- | --- | --- | --- | --- |
| Weeks 0-26 | 152 | 37 (24.3) | 156 | 32 (20.5) | 0.42 |
| Weeks 26-52 | 151 | 27 (17.9) | 154 | 32 (20.8) | 0.52 |
| Weeks 52-78 | 148 | 28 (18.9) | 154 | 31 (20.1) | 0.79 |
| Weeks 78-104 | 147 | 21 (14.3) | 153 | 22 (14.4) | 0.90 |

^a^Major medication change = start, stop or change dose of DMARD, bDMARD or glucocorticoid

^b^Percentage of patients remaining in trial

**Table F: CACE analysis**

CACE analysis used instrumental variable regression to estimate the efficacy of RAFT in reducing RA fatigue impact compared to usual care alone. One of the assumptions of CACE analysis is exclusion restriction - the offer of the intervention does not affect the outcome. In the current study those individuals randomised to a RAFT course but who could not make the course dates and did not attend had no follow-up data collected. Thus outcome data were not available for a group of individuals who would be considered non-compliant and contribute information to the CACE analysis, whilst it also violates the exclusion restriction assumption. If individuals in the intervention arm were only considered participants if they attended the first CBT session (which could then impact their outcome) then the offer of the intervention does affect outcome.

In attempt to address this issue the two stages of the 2-stage-least-squares CACE estimation were conducted separately to allow for loss to follow-up that is dependent on adherence. This first stage regression uses all individuals randomised to predict adherence. The second stage involves only those with available outcomes but models these using the predicted values of adherence obtained from the “complete” first stage.

The results of the CACE analyses are shown below, along with the primary analysis results for comparison. The CACE analyses showed that there was a larger effect of RAFT in those who adhered to the intervention (defined as attending at least two CBT sessions) compared to the standard primary analysis effectiveness estimate of the “offer” of the intervention.

**BRAF fatigue impact at 26 weeks, primary and CACE analyses**

|  | **Coefficient [95% CI]** | ***p*-value** |
| --- | --- | --- |
| Primary Analysis | -0.59 [-1.11, -0.06] | 0.03 |
| CACE | -0.69 [-1.30, -0.07] | 0.03 |

It should be noted that in the current study due to the exclusion of individuals who did not attend the first CBT session there is no one correct ideal approach; as such the results of the CACE analysis should be interpreted as part of a general sensitivity analysis and as exploratory in nature.

**Table G: Repeated measures analysis of BRAF-NRS Impact at 26, 52, 78 and 104 weeks**

|  | **26 weeks n=308** | | **52 weeks**  **n=305** | | **78 weeks**  **n=300** | | **104 weeks**  **n=296** | | **Repeated measures**  **n=308** | |
| --- | --- | --- | --- | --- | --- | --- | --- | --- | --- | --- |
| **BRAF-NRS**  **Impact** | **n** | **Mean**  **(SD)** | **n** | **Mean**  **(SD)** | **n** | **Mean**  **(SD)** | **n** | **Mean**  **(SD)** | **Adjusted mean**  **difference^a^**  **[95% CI]** | ***p*-value** |
| Control | 152 | 6.36 (2.42) | 151 | 6.38 (2.19) | 146 | 6.38 (2.23) | 145 | 6.05 (2.14) | -0.49  [-0.83, -0.14] | 0.01 |
| RAFT | 156 | 5.74 (2.41) | 154 | 5.72 (2.23) | 154 | 6.17 (2.24) | 151 | 5.54 (2.28) |  |  |

^a^Adjusted for baseline score and centre

**Table H: Change in BRAF-NRS Impact at 26 weeks for consecutive RAFT cohorts (1-4)**

|  | **Control** | | | | **RAFT** | | | |  |
| --- | --- | --- | --- | --- | --- | --- | --- | --- | --- |
|  |  | **0 weeks** | **26 weeks** | **Mean**  **Change**  **[95% CI]** |  | **0 weeks** | **26 weeks** | **Mean**  **Change**  **[95% CI]** | **Difference in**  **mean change** |
|  | **n** | **Mean (SD)** | **Mean (SD)** |  | **n** | **Mean (SD)** | **Mean (SD)** |  |  |
| 1 | 39 | 7.56 (1.31) | 6.72 (1.92) | -0.85  [-1.43, -0.26] | 37 | 6.95 (1.70) | 5.73 (2.58) | -1.22  [-2.17, -0.26] | -0.37 |
| 2 | 39 | 7.54 (1.54) | 6.44 (2.82) | -1.10  [-1.90, -0.30] | 38 | 7.21 (1.58) | 5.76 (2.62) | -1.45  [-2.34, -0.56] | -0.35 |
| 3 | 39 | 6.69 (1.81) | 6.15 (2.62) | -0.54  [-1.45, 0.37] | 42 | 7.26 (1.89) | 6.31 (2.42) | -0.95  [-1.77, -0.13] | -0.41 |
| 4 | 35 | 7.11 (1.75) | 6.09 (2.27) | -1.03  [-1.76, -0.29] | 39 | 6.95 (1.50) | 5.10 (1.90) | -1.85  [-2.51, -1.18] | -0.82 |

**Table I: Adjusted mean difference between arms for fatigue outcomes at 10 and 18 weeks (consolidation session occurred week 14)**

|  | **Week 10^a^** | | | **Week 18^b^** | | |
| --- | --- | --- | --- | --- | --- | --- |
|  | **Adjusted mean difference^a^**  **[95% CI]** | ***p*-value** | **Effect**  **size** | **Adjusted mean difference^c^**  **[95% CI]** | ***p*-value** | **Effect size** |
| BRAF-NRS Impact (0-10) | -0.51 [-0.97, -0.05] | 0.03 | 0.31 | -0.64 [-1.11, -0.17] | 0.01 | 0.39 |
| BRAF-NRS Severity (0-10) | -0.04 [-0.52, 0.45] | 0.88 |  | -0.32 [-0.81, 0.18] | 0.21 |  |
| BRAF-NRS Coping (0-10)^d^ | 0.46 [-0.05, 0.98] | 0.08 |  | 0.75 [0.20, 1.29] | 0.01 | 0.36 |
| BRAF-MDQ Overall Impact (0-70) | -2.22 [-5.00, 0.57] | 0.12 |  | -2.93 [-6.09,0.22] | 0.07 |  |
| BRAF-MDQ Physical (0-22) | -0.64 [-1.65, 0.36] | 0.21 |  | -0.89 [-1.96, 0.18] | 0.10 |  |
| BRAF-MDQ Emotional (0-12) | -0.76 [-1.44, -0.08] | 0.03 | 0.24 | -0.87 [-1.57, -0.18] | 0.01 | 0.27 |
| BRAF-MDQ Cognitive (0-15) | -0.34 [-1.06, 0.38] | 0.36 |  | -0.74 [-1.53, 0.06] | 0.07 |  |
| BRAF-MDQ Living with (0-21) | -0.55 [-1.55, 0.45] | 0.28 |  | -0.44 [-1.56, 0.68] | 0.44 |  |

^a^Control n=103-153; RAFT n=137-155; ^b^Control n=137-151; RAFT n=137-154; ^c^Linear regression adjusted for baseline outcome scores and for centre; ^d^Higher score = better outcome

**Table J: RA Self-Efficacy (RASE) item changes 0-26 weeks (n=293)**

|  | **Control**  **n=142** | **RAFT**  **n=151** |  |  |
| --- | --- | --- | --- | --- |
| **RASE item^a^** | **Mean**  **change** | **Mean**  **change** | **Difference**  **in mean change** | ***p*-value** |
| Use relaxation techniques to help with pain | -0.07 | 0.11 | 0.18 | 0.10 |
| Think about something else to help with pain | 0.04 | 0.16 | 0.12 | 0.34 |
| Use my joints carefully to help with pain | 0.20 | 0.18 | -0.02 | 0.87 |
| Think positively to help with pain | 0.06 | 0.23 | 0.17 | 0.16 |
| Avoid doing things that cause pain | 0.25 | -0.05 | -0.30 | 0.03 |
| Wind down, relax before bed, to improve sleep | 0.10 | 0.17 | 0.07 | 0.56 |
| Hot drink before bed to improve sleep | 0.14 | 0.19 | 0.05 | 0.69 |
| Use relaxation before bed to improve sleep | -0.11 | 0.17 | 0.28 | 0.02 |
| Pace myself, take RA into account to deal with fatigue | -0.08 | 0.33 | 0.41 | <0.01 |
| Accept fatigue as part of my arthritis | 0.07 | 0.40 | 0.33 | 0.01 |
| Use gadgets to help with mobility, tasks, personal care | -0.01 | 0.19 | 0.20 | 0.06 |
| Ask for help to deal with difficulties of doing everyday things | -0.01 | 0.25 | 0.26 | 0.02 |
| Do exercises to deal with difficulties of doing everyday tasks | 0.01 | 0.09 | 0.08 | 0.50 |
| Plan/prioritise to deal with difficulties doing everyday tasks | -0.01 | 0.17 | 0.19 | 0.07 |
| Educate family/friends about my RA to help relationships | 0.01 | 0.15 | 0.14 | 0.25 |
| Explain to friends and family when I do or do not need help | -0.02 | 0.04 | 0.06 | 0.61 |
| Discuss any problems with my partner or family | 0.00 | 0.10 | 0.10 | 0.38 |
| Make time for leisure activities, hobbies or socializing | -0.01 | 0.05 | 0.05 | 0.58 |
| Save energy for leisure activities, hobbies or socializing | 0.08 | 0.14 | 0.06 | 0.66 |
| Focus on the positive when I am feeling down | 0.01 | 0.15 | 0.14 | 0.21 |
| Use relaxation to deal with worries | 0.07 | 0.03 | -0.04 | 0.75 |
| Allocate time for relaxation | -0.05 | 0.04 | 0.09 | 0.39 |
| Use relaxation tape or instructions to help me relax | -0.22 | 0.18 | 0.40 | <0.01 |
| Use regular exercise | 0.02 | 0.14 | 0.12 | 0.27 |
| Be aware of my limits in exercise | -0.07 | 0.05 | 0.11 | 0.23 |
| Manage medication, knowing how and when to take it | -0.01 | 0.07 | 0.07 | 0.42 |
| Look out for and avoid side-effects of medication | -0.08 | 0.01 | 0.09 | 0.42 |
| Seek help with persistent side-effects | -0.04 | 0.00 | 0.04 | 0.66 |

^a^High score means greater self-efficacy (1-5)
